# Cytokine Receptor-Like Factor 1 (*CRLF1*) Protects against 6-Hydroxydopamine Toxicity Independent of the gp130/JAK Signaling Pathway

**DOI:** 10.1371/journal.pone.0066548

**Published:** 2013-06-20

**Authors:** Brendan D. Looyenga, James Resau, Jeffrey P. MacKeigan

**Affiliations:** 1 Laboratory of Systems Biology, Van Andel Research Institute, Grand Rapids, Michigan, United States of America; 2 Program in Biospecimen Science, Van Andel Research Institute, Grand Rapids, Michigan, United States of America; Hertie Institute for Clinical Brain Research and German Center for Neurodegenerative Diseases, Germany

## Abstract

Oxidative stress is an important cause of cellular toxicity in the central nervous system and contributes to the pathology associated with neurodegenerative disorders including Parkinson’s disease. As such, elucidation of cellular mechanisms that enhance neuronal resistance to oxidative stress may provide new avenues for therapy. In this study we employed a simple two-state cellular model to identify genes that are associated with resistance to oxidative stress induced by 6-hydroxydopamine (6-OHDA). In this model, undifferentiated neuroblastoma cells display higher sensitivity to 6-OHDA than differentiated cells. By comparing the gene expression between these two states, we identified several genes whose expression is altered concomitant with changes in 6-OHDA sensitivity. This gene set includes cytokine receptor-like factor 1 (*CRLF1*), which is up-regulated during the differentiation process and has been previously implicated in neuroprotection. We show that the product of this gene is both necessary and sufficient for increased resistance to 6-OHDA in differentiated neuroblastoma cells, and that CRLF1 serves its protective role by a cell autonomous mechanism that is independent from its known role as a co-ligand for the ciliary neurotrophic factor receptor. These data provide an additional role for CRLF1 that could potentially explain its broad expression pattern and effects on cells lacking expression of this receptor.

## Introduction

Oxidative stress plays a central role in neuronal toxicity associated with a wide variety of neurodegenerative conditions including Alzheimer’s disease, amyotrophic lateral sclerosis, Huntington’s disease and Parkinson’s disease (PD) [1]. Multiple causes of oxidative stress have been implicated in the etiology of these diseases, including both endogenous and exogenous sources. The most prominent endogenous sources of oxidative stress are mitochondria, which generate reactive oxygen species (ROS) as a byproduct of oxidative metabolism. Defects in mitochondrial function that result in aberrantly high levels of oxidative stress have been implicated in hereditary and sporadic PD, and are also associated with the normal aging process in long-lived cells such as neurons [1], [2], [3]. Such defects are exacerbated by exogenous sources of oxidative stress such as pesticides or other environmental toxins, many of which inhibit mitochondrial electron transport and further interrupt mitochondrial function [4], [5]. In the case of PD, these insults particularly affect midbrain neurons that produce the oxidizing neurotransmitter dopamine, which raises the basal threshold of oxidative stress and makes these cells especially vulnerable to transient bursts of ROS [6], [7], [8].

One of the best characterized models of oxidative stress-induced injury to dopaminergic neurons is the 6-hydroxydopamine (6-OHDA) model [9], [10]. Injection of 6-OHDA into the striatum of laboratory animals produces acute parkinsonism characterized by defects in dopamine secretion and progressive loss of tyrosine hydroxylase (TH) positive neurons in the substantia nigra [11]. Although these two features are temporally and mechanistically distinct, both appear to result from increased oxidative stress in the cytosol of dopaminergic neurons that can be offset by treatment with various natural or synthetic antioxidants [12], [13]. Because this model offers temporally well-defined and reproducible defects in the nigro-striatal system, it is has become an effective model for validation of neuroprotective compounds *in vivo*.

The 6-OHDA model has also been recapitulated *in vitro* with primary or immortalized neuron cultures [14]. Transformed neuroblastoma cell lines–in particular SH-SY5Y and SK-N-SH cells–have been broadly utilized as an experimental model for dopaminergic neuron toxicity in this context [15], [16], [17]. When differentiated *in vitro*, these cells share several features with dopaminergic neurons that make them a suitable model for neurotoxicity studies, including: 1) the ability to undergo proliferative arrest; 2) expression of the biosynthetic enzymes required for dopamine synthesis; and 3) extension of synaptically competent neurite outgrowth [17], [18]. Unlike primary dopaminergic neurons, however, immortalized neuroblastoma cells can be cultured and differentiated in pure cultures free of glial support cells, making them a more precise model for *in vitro* genetic manipulation and gene expression studies.

Previous reports have suggested that differentiation of SH-SY5Y cells changes their susceptibility to oxidative stress [19]. Since differentiation also leads to measurable changes in gene expression, the differentiation process provides a suitable two-state, “on-off” model for identifying neuroprotective genes whose expression is altered during differentiation. In this study we took advantage of the differential sensitivity of undifferentiated and differentiated neuroblastoma cell lines to 6-OHDA to identify endogenous sources of neuroprotection. Comparative analysis of gene expression between these two cellular states identified cytokine receptor-like factor 1 (*CRLF1*) as a putative mediator of oxidative stress resistance.

## Materials and Methods

### Cell Culture

SH-SY5Y and SK-N-SH neuroblastoma cell lines were obtained from American Type Culture Collection (ATCC, Manassas, VA) and cultured in OptiMEM media (Invitrogen, Calsbad, CA) containing 10% fetal bovine serum (FBS) on tissue-culture treated plates under standard growth conditions of 5% CO_2_. The undifferentiated condition was simulated by maintaining cells plated to specific densities in Neurobasal-A media (NBA, Invitrogen) containing 10% FBS. Differentiated conditions were simulated either by maintaining cells for six days in serum-free NBA containing B27 Supplement minus antioxidants (NBA/B27, Invitrogen) and 10 µM trans retinoic acid (RA), or by maintaining cells for three days in NBA/B27 with 10 µM RA and then a subsequent three days in NBA/B27 containing 100 nM 12-*O*-tetradecanoylphorbol-13-acetate (TPA). For all 96-well assays, cells were plated at a density of 2500 cells per well and allowed to adhere for 16–24 hours prior to treatment or differentiation.

### Production of shRNA and cDNA Lentiviral Stable Lines

Lentiviral plasmids (TRC collection v1, pLK0.1 vector) containing shRNAs targeted to *CRLF1* (shRNA #1–5, TRCN0000061483 thru TRCN0000061487) were obtained from Open Biosystems (Lafayette, CO). Open reading frames for CRLF1-FL or CRLF1-ΔN were cloned into the pCDH-EF1-MCS-IRES-neo lentiviral vector (System Biosciences, Mountain View, CA) for cDNA expression.

Both sets of plasmid vectors were transfected into 293FT packaging cells along with third generation packaging helper vectors (pLP1, pLP2 and pVSVG). DMEM media containing 10% FBS was removed and replaced 24 hours after transfection and then left on the producer cells for an additional 48 hours. Conditioned media containing viral particles was filtered through 0.45 µm syringe filters to remove cellular debris and frozen at −80°C in 1 mL aliquots until use.

Stable SH-SY5Y cell lines were created by infecting cells in 6 cm plates with viral conditioned media diluted 1∶3 with OptiMEM media containing 10% FBS and 8.0 µg/mL polybrene (Sigma). 48 hours post-infection, cells were passaged to 10 cm plates and selected with either puromycin (2.0 µg/mL, shRNA lines) or G418 (500 µg/mL, cDNA lines) for an additional 72–96 hours to eliminate uninfected cells. Stable lines were routinely used for all assays within 1 week of selection to eliminate artifacts caused by random selection for shRNA or cDNA inactivation. All lentiviral work was performed in a UV-sterilized biosafety cabinet under BL2 biosafety conditions after approval of the Van Andel Institute recombinant DNA committee.

### Antibodies

Mouse monoclonal antibodies to βIII tubulin (Tuj1) and gp130 (neutralizing) were obtained from R&D Systems (Minneapolis, MN). Mouse monoclonal antibodies for NeuN and NSE and the rabbit polyclonal antibody to TH were purchased from Millipore (Billerica, MA). The rabbit polyclonal antibody to MAPT/Tau and the mouse monoclonal antibody to α-tubulin were purchased from Sigma-Aldrich (St. Louis, MO). Phospho-specific and total antibodies (all rabbit polyclonal) for STAT1, STAT3, AKT, ERK, S6 and β-actin were obtained from Cell Signaling Technologies (Danvers, MA). The mouse monoclonal antibodies to CRLF1 and Hsp60 were obtained from Santa Cruz Biotechnologies (Santa Cruz, CA) and BD Biosciences (Franklin Lakes, NJ) respectively. The mouse monoclonal antibody to the V5 epitope tag was obtained from Invitrogen.

### Immunocytochemical Staining and Microscopy

Cells were seeded to coverslips and allowed to adhere for 16–24 hours prior to differentiation with RA or RA/TPA. Cells were then fixed with 4% paraformaldehyde and permeabilized with 0.2% TritonX-100 in PBS. After blocking with 5% normal goat serum in PBS, the coverslips were incubated at 4°C overnight with a 1∶1000 dilution of mouse monoclonal Tuj1 antibody and a 1∶200 dilution of rabbit polyclonal TH antibody. After washing in PBS/0.02% TritonX-100, coverslips were incubated for one hour with AlexaFluor-488 coupled anti-mouse and AlexaFluor-546 coupled anti-rabbit secondary antibodies. After a final round of washing, cells were co-stained with Hoechst 33342 to detect nuclei and coverslips were mounted on glass slides with Fluoro-gel mounting medium (Electron Microscopy Science, Hatfield, PA). Images were obtained using a Nikon Ti-E inverted fluorescence microscope equipped with DAPI, FITC and Texas Red filter sets, and processed using the NIS Elements software package (Nikon Instruments, Melville, NY).

### Immunoblotting

Cells grown in the indicated culture conditions were washed with cold PBS and harvested on ice in cold pH 7.5 lysis buffer (20 mM Tris-HCl, 150 mM NaCl, 1 mM Na_2_EDTA, 1 mM EGTA, 2.5 mM sodium pyrophosphate, 1 mM β-glycerophosphate, 50 mM sodium fluoride, 1 mM Na_3_VO_4_, 1% Triton-X100, 1 mM DTT) supplemented with protease inhibitor cocktail (Sigma-Aldrich). Soluble protein from lysates was quantified by Bradford assay (Bio-Rad, Hercules, CA). After normalization of concentration, samples were diluted with Laemmli buffer and denatured by boiling. Samples were then separated on Tris-glycine polyacrylamide gels and transferred overnight to nitrocellulose membranes in a wet transfer apparatus (Hoefer, Holliston, MA). Membranes were blocked in 3% non-fat dry milk in Tris-buffered saline/0.1% Tween (TBS-T) and probed with primary antibodies overnight at 4°C. After washing in TBS-T buffer and incubation with a horseradish peroxidase-coupled secondary antibody, membranes were incubated in enhanced chemiluminescent reagent, exposed to film and developed for signal using an *X-omat* processing machine (Kodak, Rochester, NY).

### Proliferation Assays

Cells were plated at a fixed density of 2500 cells per well to 96-well plates and allowed to adhere overnight. Media was then removed and replaced with NBA+FBS, NBA/B27+RA or NBA/B27+RA/TPA as indicated above for six days. At the end of the differentiation protocol, media was removed and cells were washed once with PBS and frozen at −80°C with 100 µL of CyQuant lysis buffer (Invitrogen) containing the CyQuant DNA intercalating fluorescent dye. Each plate was then thawed and total fluorescence was measured using a clear-bottom assay plate and an Envision multi-function plate reader (Perkin-Elmer, Waltham, MA). Replicate values were averaged and normalized to undifferentiated plating control conditions.

### 6-OHDA Toxicity Assays

Cells were plated at a fixed density of 2500 cells per well to 96-well plates and allowed to adhere overnight. Media was then removed and replaced with NBA+FBS, NBA/B27+RA or NBA/B27+RA/TPA as indicated above for six days in 100 µL per well volumes. At the end of the differentiation protocol, 10 µL of 10× concentration 6-hydroxydopamine (6-OHDA, Sigma-Aldrich) relative to the indicated final concentration was added to each well, mixed by shaking and allowed to incubate with cells for 24 hours. At the end of the incubation, media was removed and cell viability was quantified by luminescent assay using Cell Titer Glo reagent (Promega, Madison, WI). Replicate values were averaged and normalized to untreated controls (no 6-OHDA) for each different media condition used in each experiment.

For assays in which conditioned media was compared to fresh media in toxicity assays, naïve/undifferentiated cells were plated at 2500 per well in OptiMEM media with 10% FBS and allowed to adhere for 16–24 hours. Media was then removed by inverted shaking and replaced with fresh or conditioned media from the same cell type containing the indicated concentration of 6-OHDA. After 24 hours of incubation under normal TC conditions, cell viability was measured and normalized as indicated above.

### Statistical Analysis

Statistical analysis of 6-OHDA toxicity assays and generation of LD50 dose-response curves was performed with the Sigma Plot 12 software package (Systat Software, Chicago, IL). Data from each assay were fit to standard four parameter, nonlinear logistic regression curves using a dynamic fit option of 200 iterations to obtain curves with R-squared values ≥0.95 for all experiments. Significant differences between LD50 values for different experiments were established by using a two-sample t-test to determine *p*-values. LD50 values, standard errors and *p*-values for replicate experiments derived from these analyses are displayed beneath each graph in the figures.

### Gene Expression Microarray Analysis

The human gene expression microarrays were performed at the Core Laboratory of Microarray Technology at the Van Andel Research Institute with whole human genome 4×44 k gene expression microarrays from Agilent Technologies (Santa Clara, CA) to obtain the global gene profiles. This array covers 19,596 different RNA sequences from the Entrez database. Total mRNA was harvested from cells grown on 10 cm plates under the indicated treatment conditions using the RNeasy miniprep kit according to manufacturer protocol (Qiagen, Valencia, CA). RNA was quantified by UV-spectrophotometry and normalized for input of 5 µg of total RNA into each cDNA synthesis reaction. Each test sample was fluorescently labeled by Cy5, while control Universal Human Reference RNA (Stratagene, La Jolla, CA) was labeled with Cy3. Both test sample and control were hybridized together onto each array according to Agilent standard microarray procedures. After hybridization for 17 hrs at 65°C at 10 rpm, the arrays were washed and scanned with the Agilent scanner. Probe features were extracted from the microarray scan data using Feature Extraction software (Agilent Technologies).

Fluorescent intensity values for each probe were normalized to negative control probes on each array and imported into the SpotFire software program for generation of relative expression values and the heat map display (Tibco, Palo Alto, CA). Log expression of each gene was determined relative to the fluorescent intensity values from the reference RNA library. Relative changes in gene expression (Δ) in the differentiated (RA/TPA treated) versus undifferentiated (FBS treated) states were calculated by [Δ = Log_Undiff_ – Log_Diff_] for each gene. The change in gene expression (Δ) for each cell line were then plotted against each other to identify genes whose expression coordinately changes in both lines upon differentiation.

### Quantitative Reverse-Transcription Polymerase Chain Reaction (qRT-PCR)

Total mRNA was harvested from cells grown under the indicated treatment conditions and quantified as indicated above. Template cDNA was synthesized from 1.0 µg of total RNA using the iScript-Select kit and poly-dT primers (Bio-Rad) according to standard manufacturer protocol with a 90 minute extension phase to optimize synthesis of long transcripts. The products of each cDNA synthesis reaction were diluted 1∶5 in Tris-EDTA buffer and used as template for quantitative PCR. PCR reactions for each sample contained 10 µL of 2x SYBR green reaction mix (Roche Applied Science, Indianapolis, IN), 5 µL of template cDNA, 1.0 µM primers (IDT, Coralville, IA) and sterile deionized water to a final 20 µL volume. Reactions were performed on a 7500 Real Time thermocycler (Applied Biosystems, Foster City, CA) according to standard protocol with an added melting curve phase to ensure a single PCR product was detected in each well. All reactions were performed in triplicate and normalized to averaged triplicate measurements of the housekeeping gene *RPL13A*. Primers for these genes are included in the online supplement.

### Direct ELISAs

Direct enzyme-linked immunosorbent assays (ELISAs) were performed by incubating 72 hour conditioned media harvested from cultured cells on high binding-capacity 96-well plates (Invitrogen) for 16–20 hours at 4°C. After blocking in 3% non-fat dry milk and washing with PBS/0.05% Tween-20, the plates were probed with primary antibodies to CRLF1 (Santa Cruz mouse monoclonal, 1∶100) or CLCF1 (R&D Systems mouse monoclonal, 1∶200) for an additional 16–20 hours at 4°C. The following day plates were washed again and probed with anti-mouse-HRP secondary antibody (1∶1000) for 1 hour at room temperature. After a final washing step, the plate was developed with equal volumes of 3,3′,5,5′-tetramethylbenzidine substrate (TMB, Cell Signaling) and stop solution (0.16 M sulfuric acid), then measured on a plate reading spectrophotometer at 450 nm. All reactions were performed in quadruplicate and quantified relative to a standard curve of heterodimeric recombinant CRLF1/CLCF1 (R&D Systems) diluted in serum-free cell culture media. Data from this standard curve indicated that the ELISA assay was linear from 0.5 to 50 ng/mL with R^2^ = 0.994 and 0.986 for CRLF1 and CLCF1, respectively.

## Results

### Cell Culture Model of Neural Differentiation

Several distinct cell culture paradigms for differentiating neuroblastoma cells into a neural lineage have been reported [18], [20], [21]. The success of these paradigms can be evaluated by three parameters: 1) induction of neurite outgrowth; 2) up-regulation of neural-specific differentiation markers; and 3) induction of mitotic arrest. Long-term (six days or more) treatment of neuroblastoma cells with trans-retinoic acid (RA) in serum-free conditions effectively meets all of these criteria, and is the most commonly used method of differentiation in this model system [17], [20], [21]. This method can be enhanced by addition of the phorbol ester 12-O-tetradecanoylphorbol-13-acetate (TPA), which increases the number and complexity of neurite processes in differentiating cells [18], [20].

**Figure 1:**
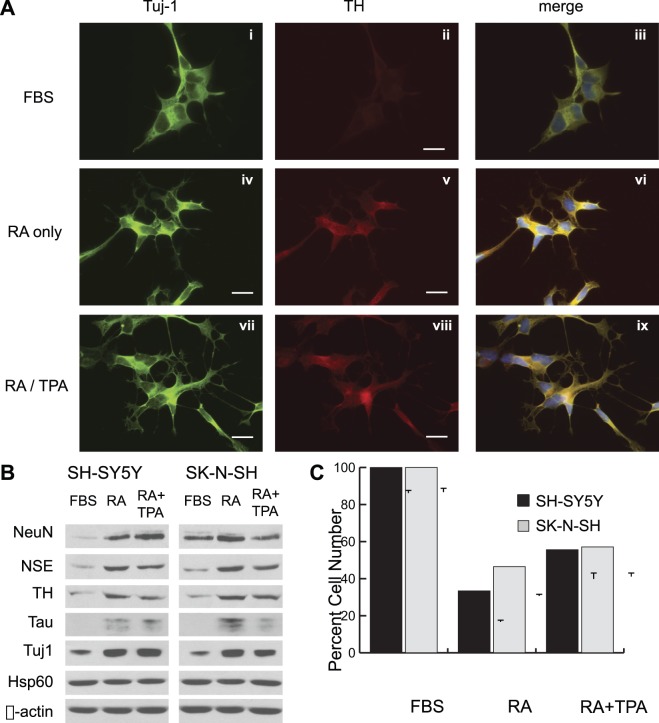
Differentiation of neuroblastoma cell lines by combined treatment with retinoic acid and TPA. **A**, SH-SY5Y neuroblastoma cells were plated to coverslips and treated for six days with Neurobasal-A (NBA) medium containing 10% fetal bovine serum (FBS, panels i-iii) or serum-free NBA/B27 medium containing 10 µM retinoic acid (RA, panels iv-vi), or for three days with RA followed by three days of NBA/B27 containing 100 nM TPA (panels vii-ix). Cells were fixed and immunostained for the pan-neuronal marker β-III tubulin (Tuj-1, green) and the dopaminergic neuron marker tyrosine hydroxylase (TH, red). Nuclear co-stain was performed using Hoechst 33342 (blue). **B**, Protein lysates were extracted from SH-SY5Y or SK-N-SH neuroblastoma cells treated as in A. Equal protein amounts for each condition were separated by SDS-PAGE and analyzed by immunoblot using the indicated antibodies. Hsp60 and β-actin were used as controls to demonstrate equal loading. **C**, Cells were plated to 96-well plates at a fixed density and allowed to adhere for 24 hours. Replicate wells were then treated for six days as in A, and relative cell number was analyzed by cellular DNA content and normalized to cells cultured in normal undifferentiated conditions. A decrease in proliferative rate under differentiation conditions is indicated by the reduced cell number. Error bars indicate standard deviation in replicate (n = 6) samples.

To validate the latter strategy, we treated SH-SY5Y neuroblastoma cells with 10 µM RA in serum-free media for three days, and then exchanged the RA for 100 nM TPA for an additional three days. Alternatively, we treated cells continuously with RA in serum-free media for six days with media exchange after the first three days. We then compared immunocytochemical staining for two markers of neural differentiation–βIII-tubulin (Tuj1) and tyrosine hydroxylase (TH)–in cells kept in complete media with fetal bovine serum (FBS) or in cells treated under these two conditions indicated above (Fig. 1A). While Tuj1 stains undifferentiated cells, TH is almost completely absent prior to differentiation (Fig. 1A, panels i-iii). However, staining for both markers increases in intensity upon stimulation with RA or RA/TPA (Fig. 1A, panels iv-ix). In addition, Tuj1 staining reveals extension of neurites during differentiation, which increase in number and complexity compared to undifferentiated cells (Fig. 1A, panels vii-ix).

To further validate that RA and RA/TPA treatment induce neuronal differentiation of neuroblastoma cell lines, we performed immunoblots for five markers of neuronal differentiation on lysates from SH-SY5Y and SK-N-SH cells treated as indicated above (Fig. 1B). As previously indicated, both Tuj1 and TH increase during differentiation, as do the markers for nuclear neuronal protein (NeuN) and neuron-specific enolase (NSE). The increase in the microtubule-associated protein Tau, which stabilizes microtubule bundles in neurite extensions, is consistent with extension and maturation of neurites seen in Tuj1 stained cells. In contrast to these markers, expression of β-actin and the mitochondrial chaperone Hsp60 are unchanged during the differentiation process.

Finally, we also determined the relative number of cells in culture after six days of treatment with media containing FBS or RA to assess whether proliferative arrest was occurring during the differentiation process. As expected, serum-withdraw and treatment with RA reproducibly led to a ∼60% decrease in cell number, while combined treatment with RA/TPA produced a 50% decrease in cell number for both neuroblastoma cell lines (Fig. 1C). Collectively, these data demonstrate that treatment of neuroblastoma cells with RA or RA/TPA produces all of the phenotypes consistent with neuronal differentiation.

### Differentiation Alters Sensitivity of Neuroblastoma Cells to 6-OHDA in Cell Autonomous Fashion

**Figure 2:**
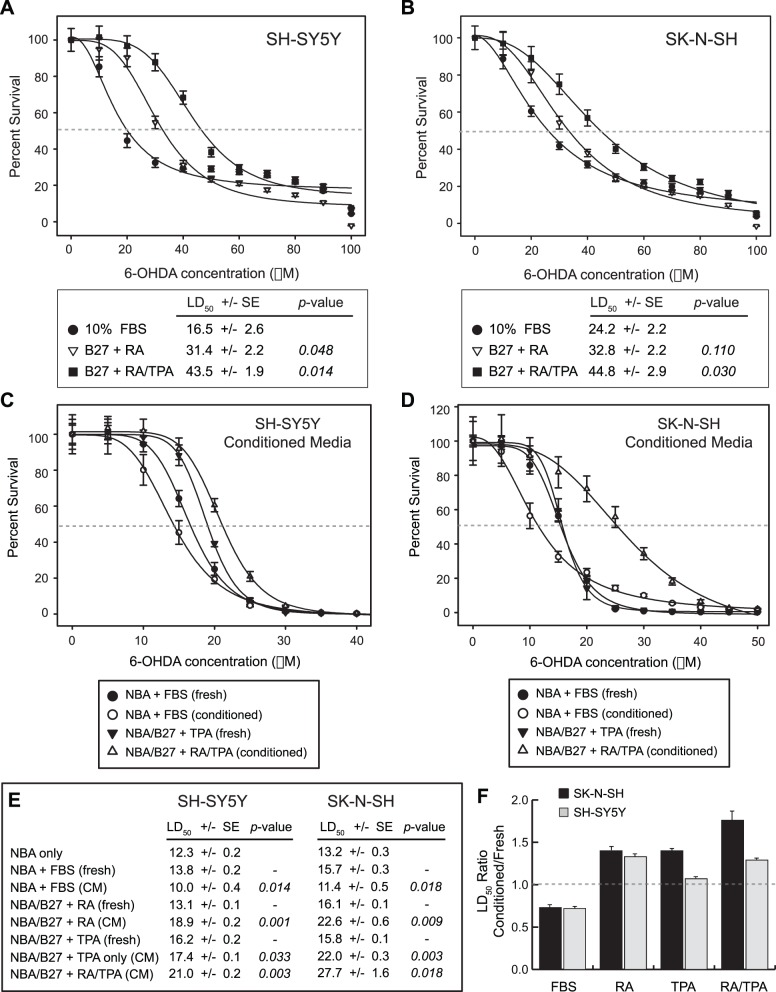
Differentiation of neuroblastoma cells promotes resistance to 6-OHDA toxicity. **A**, SH-SY5Y cells were plated and allowed to adhere overnight, then differentiated with 10 µM retinoic acid only (six days) or with retinoic acid and 100 nM TPA (three days each). Cells were subsequently treated for 24 hours with different doses of 6-OHDA diluted in serum-free NBA/B27 media and analyzed for cellular viability. Relative cell number was normalized to untreated cells and plotted according to 6-OHDA concentration. Dose-response curves were generated to identify the LD50 (gray dashed line) of each cellular state. LD50 values ± SE are indicated in the table below the graph along with significance scores (*p*-value) comparing treated samples to untreated controls. **B**, SK-N-SH cells were treated and assayed as in A. LD50 values ± SE are indicated in the table below the graph along with *p*-values comparing treated samples to untreated controls. **C**, SH-SY5Y cells were plated and allowed to adhere overnight. They were subsequently treated with the indicated doses of 6-OHDA diluted either in fresh NBA media (alone or containing 10% FBS, 10 µM RA or 100 nM TPA), or in conditioned media harvested from SH-SY5Y cells that had been cultured in 10% FBS or differentiation media (RA only, TPA only or RA/TPA) for six days. After 24 hours the cells were analyzed for viability and relative cell number was determined relative to untreated controls. **D**, SK-N-SH cells were treated and assayed as in C. **E**, LD50 values ± SE for naïve SH-SY5Y and SK-N-SH cells cultured in fresh or conditioned media from each treatment paradigm. For all experiments, cells were treated and assayed as in C. Significance scores (*p*-value) are shown for comparisons between fresh and conditioned media (CM) for each condition. **F**, The ratio of LD50 values in conditioned versus fresh media for each media condition was calculated to determine whether media from conditioned cells contains factors that protect naïve/undifferentiated cells from 6-OHDA toxicity. Values >1 indicate a survival advantage in conditioned media, whereas values ≤1 indicate a lack of survival advantage or disadvantage in conditioned media.

Differentiation of neuroblastoma cells toward a neuronal phenotype leads to measurable changes in susceptibility to oxidative stress [17], [22]. To demonstrate this change in oxidative stress resistance, we performed dose-response survival assays on neuroblastoma cells with 6-OHDA. Undifferentiated SH-SY5Y and SK-N-SH cells cultured in media containing FBS show a rapid decline in survival in response to increasing 6-OHDA concentration, with 50% lethal dose toxicity (LD50) values of 16.5±2.6 µM and 24.2±2.2 µM, respectively (Fig. 2A–B). Differentiation over a six-day timecourse with RA or RA/TPA, however, reproducibly promotes a shift in 6-OHDA resistance. In RA only conditions, SH-SY5Y and SK-N-SH cells demonstrate LD50 values of 31.4±2.2 µM and 32.8±2.2 µM. Addition of TPA after three days further increases the LD50 values to 43.5±1.9 µM and 44.8±2.9 µM, respectively. Importantly, these changes in 6-OHDA sensitivity appear to result from a general resistance to oxidative stress rather than inhibition of mitochondrial function–which has also been ascribed to 6-OHDA [19], [23]–since differentiation of both cell lines had no effect on their sensitivity to a broad panel of mitochondrial electron transport chain inhibitors (Fig. S1).

Non-cell autonomous factors secreted by differentiated cells, which could ostensibly provide neuroprotective effects by stimulating cell survival or scavenging/detoxifying oxidative species, would be expected to protect undifferentiated/naïve neuroblastoma cells from 6-OHDA toxicity. Conversely, intracellular protective factors expressed in differentiated cells would not be secreted into the media, and would therefore not be expected to protect undifferentiated/naïve cells. To determine whether the protective effect of RA/TPA-mediated differentiation is derived from cell autonomous or non-cell autonomous factors, we performed dose-response survival assays on naïve neuroblastoma cells in culture for 24 hours with different concentrations of 6-OHDA diluted in fresh media or six-day conditioned media from cells treated with Neurobasal-A media (NBA) containing FBS, RA or RA/TPA (Fig. 2C–D, inset table, E). A protective effect of 1.4 to 1.6-fold, which was more pronounced in SK-N-SH cells, was observed for RA or RA/TPA conditioned media over fresh media of the same type, while treatment in conditioned media from cells grown in FBS actually decreased survival compared to fresh media containing FBS (Fig. 2F). These data suggest that secreted factors present in the conditioned media from differentiated cells may play a role in protection from 6-OHDA toxicity.

### Identification of Differentially Expressed Genes in Differentiated Neuroblastoma Cells

**Figure 3:**
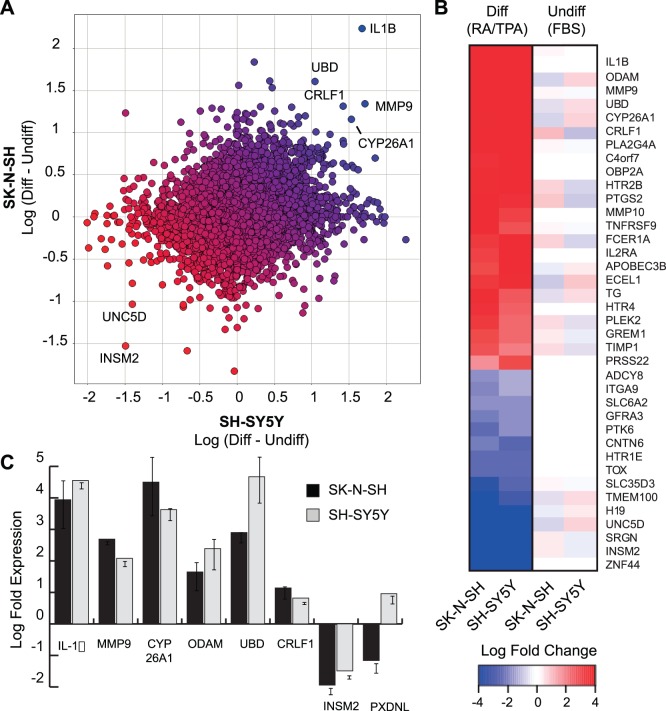
Changes in gene expression in response to RA/TPA-induced differentiation. **A**, Global gene expression in undifferentiated and differentiated SH-SY5Y and SK-N-SH cells was determined using Agilent 44 K gene expression arrays. Changes in gene expression were determined by subtracting the normalized log fold expression value of undifferentiated cells from that of differentiated cells for each line. Positive values indicate genes whose expression increase (blue), while negative values indicate genes whose expression decrease (red) in response to differentiation. The change in gene expression in SH-SY5Y cells was plotted against that of SK-N-SH cells to identify genes that were coordinately altered in both lines. **B**, Heat map illustration of genes whose expression were either significantly increased or decreased (≥1.5 fold) upon differentiation. Scale corresponds to log fold change in gene expression relative to expression control. **C**, Coordinate changes in gene expression in SH-SY5Y and SK-N-SH cells identified by expression array were validated by quantitative RT-PCR for the indicated genes. Error bars indicate standard deviation in replicate wells (n = 3).

Because the protective effect of differentiation can be recapitulated in two separate neuroblastoma cell lines, we reasoned that comparison of gene expression between undifferentiated and differentiated cells in both lines would allow us to narrow the list of potential neuroprotective factors over either cell line individually. Because protection against 6-OHDA toxicity was more pronounced in RA/TPA treated cells, we chose to compare cells in this condition to undifferentiated cells cultured in FBS for gene expression analysis. Gene expression analysis was performed using two-color hybridization to Agilent 44K microarrays, which permits normalization of gene expression signals from each cell condition to a universal human reference expression library [24]. The difference between normalized log ratio values for each gene on the array was calculated for undifferentiated versus differentiated conditions in each cell line, and is displayed as a comparison between SH-SY5Y and SK-N-SH cells (Fig. 3A). Positive values indicate genes whose expression is up-regulated during differentiation, while negative values indicate genes that are down-regulated. The most differentially expressed genes common to both cell lines are displayed with their relative log fold change (Fig. 3B). Top hits on this list include genes known to be modulated during RA-mediated neuronal differentiation, including CYP26A1 (P450 hydroxylase responsible for RA metabolism) and MMP9 (involved in neurite outgrowth) [25].

To ensure that the microarray data accurately detected changes in gene expression, we performed quantitative RT-PCR on five genes whose expression changed during RA/TPA mediated differentiation. This list includes hits that were up-regulated in both cell lines (interleukin-1-beta, *IL-1β*; matrix metalloproteinase-9, *MMP9*; odontogenic ameloblast associated, *ODAM*; ubiquitin-D, *UBD*; cytokine receptor-like factor 1, *CRLF1*), down-regulated in both lines (insulinoma-associated 2, *INSM2*) or differentially expressed between SH-SY5Y and SK-N-SH cells (peroxidasin-like homolog, *PXDNL*). As expected, analysis of gene expression by qRT-PCR correlated closely with the microarray results, but with a higher dynamic range of expression (Fig. 3C).

Results of the microarray data were analyzed using gene set enrichment analysis (GSEA) to detect patterns of coordinate gene expression that correlate with differentiation [26], [27]. The primary findings from this analysis suggested that nuclear factor kappa-B (NF-kB) and inflammatory signaling were activated upon differentiation (Fig. S2A). Because several of the top hits in our expression analysis are known targets of NF-kB (Fig. S2B), we sought to determine whether this pathway was involved in protection from 6-OHDA-mediated oxidative stress. Upon further analysis, however, we determined that activation of this pathway was induced by serum-free media conditions and not differentiation per se (Fig. S2C). Activation of NF-kB in serum-free conditions was actually dampened by both RA and TPA, suggesting that the protective effects of these compounds are not mediated by this pathway. Furthermore, treatment of neuroblastoma cells with the inflammatory cytokine interleukin-1-beta (IL-1β), a common target of NF-kB signaling and the most highly up-regulated gene in our study, failed to protect them from 6-OHDA toxicity (Fig. S2D). Together these data suggest that activation of NF-kB and inflammatory signaling during the differentiation process is unrelated to protection from 6-OHDA.

Besides those genes whose expression is directly connected to RA metabolism (CYP26A1) or NF-kB signaling (IL-1β, MMP9, UBD), the most differentially expressed genes from our microarray analysis were odontogenic ameloblast-associated protein (*ODAM*) and cytokine receptor-like factor 1 (*CRLF1*) (Fig. 3A–B). Very little is known about the function of ODAM, and it is not normally expressed in neural or proneural tissues in mammals [28]. In contrast, the product of *CRLF1* is a 43 kilodalton protein that dimerizes with cardiotrophin-like cytokine factor 1 (CLCF1) to produce a secreted ligand (CLC/CLF) belonging to the interleukin-6 family of cytokines [29], [30]. This ligand is a known neurotrophic factor whose dysfunction or loss has been implicated in a spectrum of human neurological disorders characterized by developmental delays and cold-induced sweating syndrome [31], [32], [33]. Because inhibition of signaling by the CLC/CLF receptor has previously been connected to oxidative stress, we chose to focus on CRLF1 as a potential mediator of oxidative stress resistance during differentiation of neuroblastoma cells [34].

### CRLF1 is Necessary for Protection of Differentiated Neuroblastoma Cells from 6-OHDA

**Figure 4:**
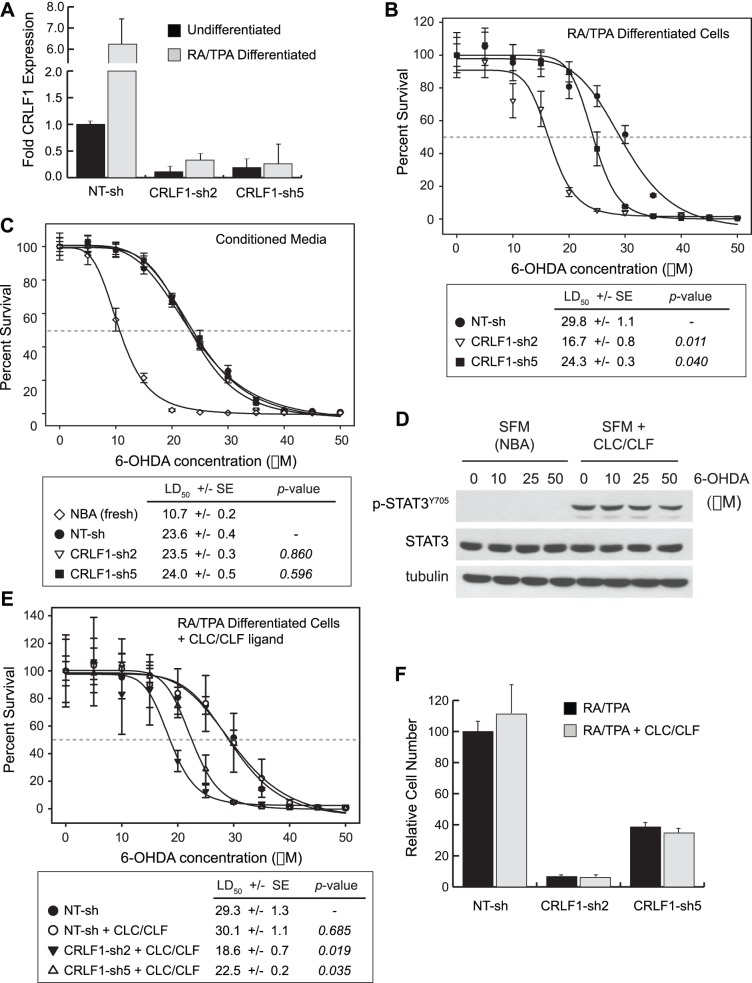
CRLF1 is required for differentiation-induced resistance to 6-OHDA independent of the CNTF receptor. A, Relative expression of CRLF1 was determined by quantitative RT-PCR in in stably selected SH-SH5Y cell lines containing control (non-targeting shRNA, NT-sh) and CRLF1-targeted shRNAs. Expression was normalized relative to basal expression in undifferentiated control cells. Error bars indicate standard deviation in replicate (n = 3) samples. **B**, Survival of the indicated stable shRNA cell lines in response to increasing doses of 6-OHDA. Dose-response curves were generated to identify the LD50 (gray dashed line) of each cellular state. LD50 values ± SE are indicated in the table below the graph along with significance scores (*p*-value) comparing CRLF1-shRNA expressing lines to NT-sh. **C**, Different concentrations of 6-OHDA diluted in conditioned media harvested from RA/TPA differentiated cells of each of the indicated stable shRNA lines were used to treated naïve SH-SY5Y cells. Dose-response curves were generated to identify the LD50 (gray dashed line) of cells treated with each media condition. LD50 values ± SE are indicated in the table below the graph along with *p*-values comparing CRLF1-shRNA expressing lines to NT-sh. **D**, Immunoblot analysis of SH-SY5Y cells treated for 15 minutes with 5 ng/mL CLC/CLF ± the indicated pre-treatment (1 hour) dose of 6-OHDA indicates that STAT3 is phosphorylated in response to the ligand independent of 6-OHDA concentration. Total STAT3 and tubulin were used as controls to demonstrate equal protein loading. **E**, Survival of the indicated stable shRNA cell lines in response to increasing doses of 6-OHDA in media ±10 ng/mL recombinant CLC/CLF ligand. LD50 values ± SE are indicated in the table below the graph along with *p*-values comparing CLC/CLF-treated cells to untreated NT-sh cells. **F**, The indicated SH-SY5Y stable shRNA lines were plated at equal densities and differentiated over six days in NBA media ±10 ng/mL recombinant CLC/CLF. Error bars indicate standard deviation in replicate (n = 6) samples.

To determine whether up-regulation of CRLF1 is required for protection of differentiated neuroblastoma cells from 6-OHDA, we employed a loss-of-function strategy in SH-SY5Y cells by identifying lentiviral short hairpin RNAs (shRNAs) that effectively decrease expression of the mRNA transcript by greater than 90% (Fig. S3A). Two of the five shRNAs (CRLF1-sh2 and sh5) are able to reduce expression of CRLF1 below that of undifferentiated cells even after six days of treatment with the RA/TPA differentiation protocol (Fig. 4A).

SH-SY5Y cells with stable integration of non-targeting control shRNA (non-targeting, NT-sh) or CRLF1 shRNAs were differentiated with RA/TPA and assayed for 6-OHDA sensitivity using the same methods as above. Compared to the control line, SH-SY5Y cells with reduced CRLF1 were significantly more sensitive to 6-OHDA (Fig. 4B). These lines displayed LD50 values of 16.7±0.8 µM and 24.3±0.3 µM in comparison to the LD50 of 29.8±1.1 µM for NT-sh cells. Because CRLF1 is primarily thought to function as a secreted factor, we expected that use of conditioned media from differentiated SH-SY5Y cells depleted of CRLF1 might provide less protection from 6-OHDA toxicity than conditioned media from control cells. Surprisingly, though, we found that conditioned media from control and CRLF1 knockdown cells were equally effective at protecting naïve SH-SY5Y cells from 6-OHDA (Fig. 4C). These data suggest that the protective role of CRLF1 either derives from long-term signaling programs associated with differentiation or from an undescribed cell-autonomous function.

To further explore the possibility that CRLF1 functions in cell autonomous fashion, we examined the effect of exogenous CLCF1/CRLF1 heterodimeric ligand (CLC/CLF) on SH-SY5Y survival. We first demonstrated that SH-SY5Y cells are competent to respond to this ligand by treating cells with a fixed dose of 5 ng/mL for 15 minutes, and then assaying for pathway activation by immunoblot. As expected, treatment of cells with CLC/CLF effectively induces the phosphorylation of STAT3, a primary effector of signaling by this ligand (Fig. 4D). The efficacy of CLC/CLF is not compromised by pre-treatment of cells with 6-OHDA, suggesting that the two stimuli do not directly interfere with each other in SH-SY5Y cells (Fig. 4D) [34].

Interestingly, combined treatment of differentiated cells with CLC/CLF (10 ng/mL) and 6-OHDA failed to increase resistance to 6-OHDA in both control and CRLF1 knockdown cell lines (Fig. 4E). Similarly, continuous treatment with recombinant CLC/CLF (10 ng/mL) over six days of differentiation was unable to rescue the basal defect in cell survival induced by CRLCF1 knockdown (Fig. 4F). Consistent with these data, we found that stable knockdown of CRLF1 in SH-SY5Y cells had no effect on STAT3 activation in the undifferentiated or differentiated state, even after treatment of cells with 6-OHDA (Fig. S3B). Knockdown of CRLF1 did, however, compromise phosphorylation of the mTOR substrate S6 in RA/TPA differentiated cells, particularly when they were treated with 6-OHDA (Fig. S3B). Though the significance of this latter finding is unclear, these data collectively suggest that the protective effect of CRLF1 in response to 6-OHDA is unrelated to its function as a co-ligand with CLCF1 and agonist of the JAK2/STAT3 pathway.

### Inhibition of Signaling through the gp-130/JAK2 Signaling Pathway Fails to Impact 6-OHDA Sensitivity

**Figure 5:**
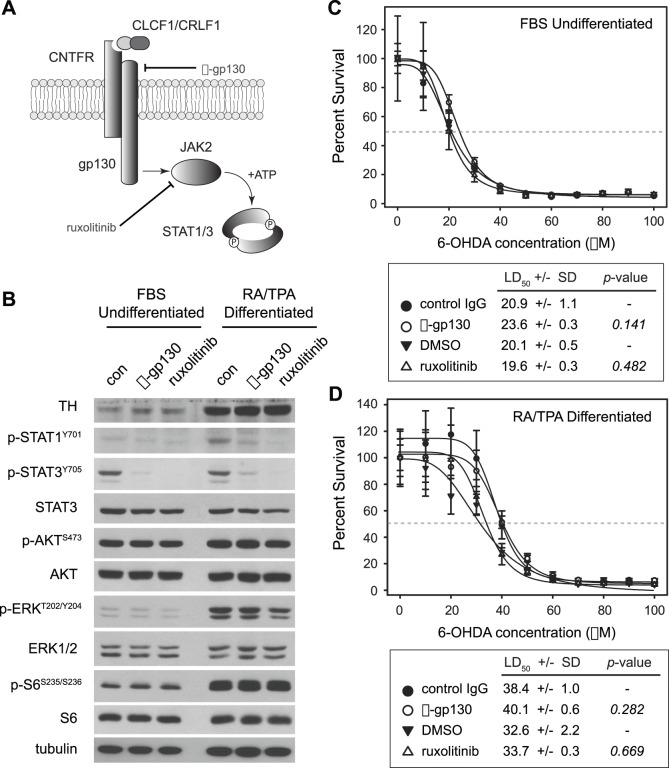
Inhibition of gp130/JAK signaling fails to affect the response of SH-SY5Y cells to 6-OHDA treatment. **A**, CLCF1/CRLF1 heterodimers are ligands for the ciliary neurotrophic factor receptor (CNTFR) complex, which includes the co-receptor gp130. This complex activates JAK family tyrosine kinases, prominently JAK2, which subsequently phosphorylate an array of targets including STAT family proteins STAT1 and STAT3. STAT proteins are dimeric transcription factors that translocate to the nucleus and regulate gene expression upon phosphorylation. **B**, Immunoblot analysis demonstrates that inhibition of the CNTFR/JAK/STAT signaling pathway with gp130 neutralizing antibodies (α-gp130) or the JAK1/2 specific inhibitor ruxolitinib (1 µM) blocks activation of STAT1 and STAT3 in both undifferentiated and RA/TPA differentiated SH-SY5Y cells compared to untreated control (con) cells of the same condition. Note that these inhibitors are highly specific for this pathway, and fail to impact growth/survival signaling by other key pathways including the MAPK (pERK), PI(3)K/AKT (pAKT) and mTOR (pS6) pathways. Control blots tyrosine hydroxylase (TH) are included to demonstrate differentiation, whereas blots for ERK1/2, AKT, S6 and tubulin are included to demonstrate equal protein loading. **C**, Undifferentiated SH-SY5Y cells cultured in media containing 10% FBS and α-gp130 (1 µg/mL), ruxolitinib (1 µM) or vehicle control (1 µg/mL non-specific mouse IgG or 0.1% DMSO) were concomitantly treated with different doses of 6-OHDA for 24 hours. Relative cell number was normalized to untreated cells and plotted according to 6-OHDA concentration. Dose-response curves were generated to identify the LD50 (gray dashed line) of each cellular state. LD50 values ± SE are indicated in the table below the graph along with significance scores (*p*-value) comparing antibody or drug-treated cells to controls. **D**, RA/TPA differentiated SH-SY5Y cells were treated with pathways inhibitors and 6-OHDA, and cell survival was analyzed as in C. LD50 values ± SE are indicated in the table below the graph along with *p*-values comparing antibody or drug-treated cells to controls. Note that neither inhibitor has a significant effect on the survival of cells in response to 6-OHDA.

Because the signaling pathway downstream of heterodimeric CLC/CLF is prominently associated with cell survival in neurons and neural progenitors, we wanted to ensure that blockade of this pathway–which could ostensibly be caused by CRLF1 knockdown–has no effect on 6-OHDA sensitivity in SH-SY5Y cells (Fig. 5A). Under normal culture conditions in media containing serum, SH-SY5Y cells demonstrate basal activation of STAT3, but not STAT1 (Fig. 5B). Differentiation of these cells with RA/TPA does not increase STAT3 activation, but does promote activation of STAT1. Treatment of SH-SY5Y cells in either culture condition with antibodies that neutralize the CLC/CLF co-receptor gp130 effectively blocks activation of both STAT1 and STAT3. Similarly, treatment with the JAK1/2 kinase inhibitor ruxolitinib (1 µM) also inhibits the activation of these proteins (Fig. 5B). Both inhibitors are highly specific for cytokine signaling, indicated by their lack of effect on other common growth factor survival pathways associated with PI-3-kinase, MAPK and mTOR (Fig. 5B).

To determine whether blockade of STAT1 and STAT3 activity affects 6-OHDA sensitivity, we treated SH-SY5Y cells with the two inhibitors for 24 hours and then performed 6-OHDA toxicity assays as before. In undifferentiated cells, neither the neutralizing gp130 antibody (1 µg/mL) nor ruxolitinib (1 µM) produce a significant change in 6-OHDA sensitivity compared to control antibody (IgG) or vehicle (DMSO) (Fig. 5C). Though differentiation of SH-SY5Y cells with RA/TPA decreased their sensitivity to 6-OHDA as before, inhibition of gp130 or JAK1/2 in this context again had no effect on their survival in response to 6-OHDA (Fig. 5D). Together these data indicate that signaling of secreted, soluble CLC/CLF through gp130 and JAK kinases is dispensible for resistance to 6-OHDA in neuroblastoma cells regardless of their differentiation state. As such, it is unlikely that the connection of CRLF1 to 6-OHDA sensitivity during neuronal differentiation is associated with its known role in CLC/CLF secretion or signaling.

### CRLF1 is Sufficient to Promote Oxidative Stress Resistance in Cell Autonomous Fashion

**Figure 6:**
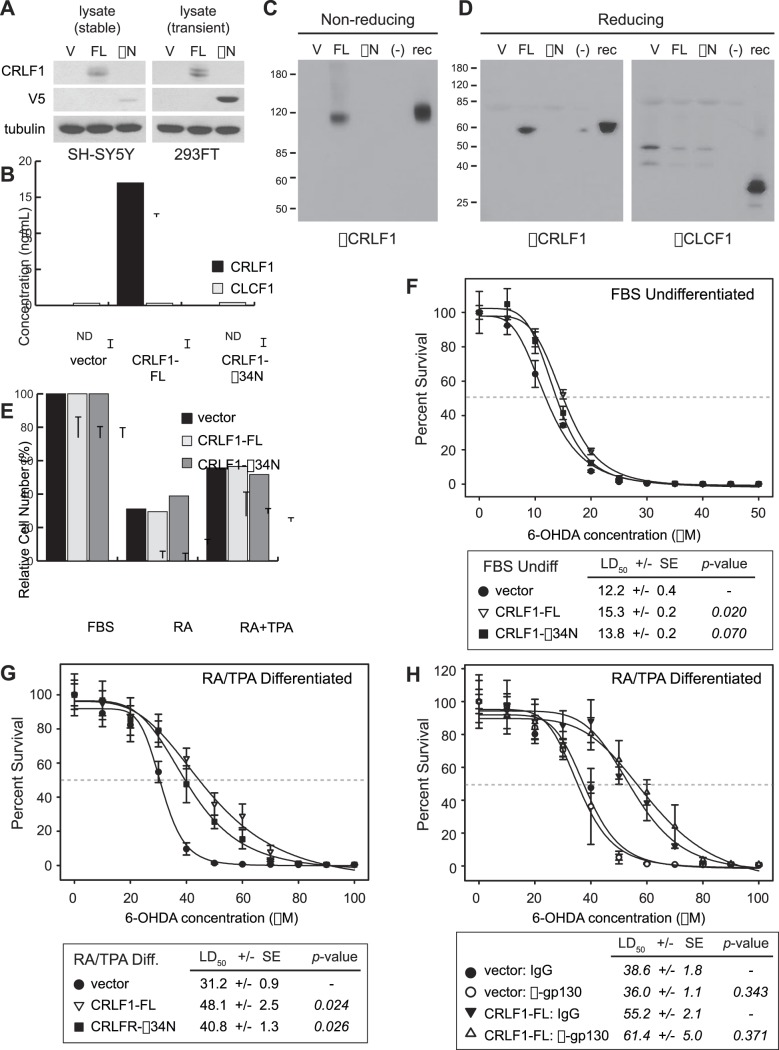
Ectopic expression of CRLF1 protects differentiated SH-SY5Y cells from 6-OHDA toxicity independent of gp130 signaling. **A**, Full-length CRLF1 (FL) and a 34 amino acid N-terminal deletion mutant (ΔN) were stably expressed from lentiviral vectors in SH-SY5Y cells or transiently expressed in 293FT cells. Expression of each protein was established by immunoblot analysis of cell lysates and conditioned media. The Δ34N mutant also contains a V5 epitope upstream of the CRLF1 sequence. **B**, Expression of CRLF1 and CLCF1 in conditioned media harvested from stable SH-SY5Y lines was determined by direct ELISA. Endogenous CRLF1 was below the limit of detection (n.d.) in vector and n-terminal truncation mutant (Δ34N) cell lines. **C,D**, Proteins precipitated from conditioned media of the indicated stable cell lines were separated by non-reducing (C) or reducing (D) SDS-PAGE, and then analyzed for CRLF1 or CLCF1 expression by immunoblot. **E**, The effect of stably expressing CRLF1-FL or CRLF1-ΔN on SH-SY5Y differentiation-induced cell cycle arrest was determined by analyzing the relative cell number of each line after six days culture in NBA media containing 10% FBS or 10 µM RA, or after three days in RA followed by three days in 100 nM TPA. Relative cell number was analyzed by cellular DNA content and normalized to undifferentiated conditions. Error bars indicate standard deviation in replicate (n = 6) samples. **F**, Survival of undifferentiated stable cell lines in response to increasing doses of 6-OHDA. Relative cell number was normalized to untreated cells and plotted according to 6-OHDA concentration. Dose-response curves were generated to identify the LD50 (gray dashed line) of each cellular state. LD50 values ± SE are indicated in the table below the graph along with significance scores (*p*-value) transgenic cells to vector controls. **G**, Survival of RA/TPA differentiated stable isogenic cell lines in response to increasing doses of 6-OHDA was determined as in F. **H**, Survival of RA/TPA differentiated stable isogenic cell lines in response to increasing doses of 6-OHDA was determined in the presence of gp130 neutralizing (α-gp130) or control (IgG) antibodies. Data was analyzed as in F.

To complement our loss-of-function data, which suggest that CRLF1 is required for differentiation-induced resistance to 6-OHDA, we created stable polyclonal lines of SH-SY5Y cells that transgenically express exogenous CRLF1 from the human elongation factor 1 (EF1) promoter. In addition to vector control cells, we created two separate transgenic lines for CRLF1 expression. The first line expresses untagged, full-length CRLF1 (CRLF1-FL), while the second line expresses a V5 epitope tagged version of CRLF1 that lacks the N-terminal 34 amino acids (CRLF1-Δ34N). This deletion mutant lacks the signal peptide for secretion and the N-terminal epitope against which the anti-CRLF1 antibody was raised, but can instead be detected with an antibody raised against the V5 epitope. As expected, we found that full-length CRLF1 could be detected in cell lysates and in conditioned media, while the CRLF1-Δ34N mutant could only be detected in cell lysates (Fig. 6A). Expression of exogenous, full-length CRLF1 in 72 hour conditioned media from CRLF1 transgenic cells was determined to be 17.0+/−0.4 ng/mL by direct ELISA (Fig. 6B).

Exogenous CRLF1 secreted from SH-SY5Y cells did not appear to be bound to CLCF1, as levels of this cytokine did not increase in parallel with CRLF1 (Fig. 6B). We confirmed this finding by separating proteins precipitated from conditioned media under non-reducing and reducing gel electrophoresis conditions. Full-length CRLF1 secreted from SH-SY5Y cells appears as a band of about 110 kilodaltons on non-reducing gels, which is slightly smaller than recombinant CLCF1/CRLF1 (Fig. 6C). Upon reduction, proteins secreted from SH-SY5Y display a 55 kilodalton CRLF1 protein band, and are negative for monomers of CLCF1, suggesting that the native 110 kilodalton band is a CRLF1 homodimer (Fig. 6D). This data is consistent with previous work in which recombinant CRLF1 expression in Sf9 or CHO cells resulted in secretion of homodimeric CRLF1 [35].

Before testing the sensitivity of the isogenic lines to 6-OHDA, we determined that the proliferation kinetics and cellular morphology associated with differentiation were unaffected by CRLF1-FL or CRLF1-Δ34N (Fig. 6E). Similarly, neither form of CRLF1 activated STAT3 above basal levels in stable SH-SY5Y cell lines or during transient expression in heterologous 293FT cells (Fig. S3B). These data collectively indicate that CRLF1 overexpression does not impact cycle regulation or signaling through the gp130/JAK2/STAT3 signaling axis in SH-SY5Y cells, and thus is unlikely to exert any protective effects via these mechanisms.

To further determine whether CRLF1 overexpression is protective against 6-OHDA, we replicated the previous dose-response toxicity assays in the stable cell lines described above in the undifferentiated and RA/TPA differentiated states. In undifferentiated cells cultured in FBS, neither CRLF1-FL nor CRLF1-Δ34N exerted a protective effect on SH-SY5Y cells (Fig. 6F). In the RA/TPA differentiated state, however, we observed that CRLF1-FL and, to a lesser extent CRLF1-Δ34N, decreased the sensitivity of SH-SY5Y cells to 6-OHDA (Fig. 6G). Protection of differentiated SH-SY5Y cells from 6-OHDA toxicity was independent of the gp130 signaling pathway, as neutralizing antibodies directed against gp130 failed to block the protective effect of full-length CRLF1 (Fig. 6H). These data therefore suggest that secretion of CRLF1, but not binding to or activation of gp130, is required for it to exert its protective effect. This effect appears to be mediated by secretion of CRLF1 homodimers, though the receptors and signaling pathways affected by this ligand await further investigation.

## Discussion

It is now widely accepted that idiopathic forms of many neurodegenerative diseases result from interactions between environmental stressors and low-penetrance genetic variation in stress resistance genes [36]. When superimposed upon “normal” age-related deficits in cellular homeostasis, these two triggers can promote the loss or dysfunction of specific neuronal subpopulations and lead to a collection of neurological deficits associated with a specific neurodegenerative disease [3], [37]. While the precise environmental insults and genetic polymorphisms associated with each disease differ, they often impinge upon similar mechanisms at the cellular level. In particular, dysfunctions in proteomic homeostasis and mitochondrial metabolism have been repeatedly implicated in neurodegenerative disease [1], [38]. These deficits result in protein misfolding/aggregation and oxidative stress, respectively, both of which are highly toxic to long-lived, quiescent cells such as neurons.

In this study we chose to focus on the regulation of endogenous oxidative stress resistance in a simplified genetic model of neuroprotection by correlating changes in gene expression to 6-OHDA resistance in SH-SY5Y cells. This approach allowed us to identify *CRLF1* as a potential oxidative stress resistance gene in neurons. The protective function we identified appears to be specific to the differentiated state of SH-SY5Y cells, consistent with *CRLF1* being a neuroprotective gene. Most surprising was our finding that the protein product of this gene appears to be protective in cell autonomous fashion. Our data suggest a new role for CRLF1 that is mechanistically distinct from its previously discovered role as a co-ligand for CNTFR and agonist of the gp130/JAK/STAT signaling pathway [29], [30]. Because inhibition of this pathway by pharmacologic means clearly has no effect on SH-SY5Y resistance to 6-OHDA, we conclude CRLF1 has secondary functions independent of acting as a secreted ligand for CNTFR.

Naturally occurring mutations to *CRLF1* are associated with a spectrum of neurological disorders including type I cold induced sweating syndrome 1 (CISS1, OMIM no. 272430) and Crisponi syndrome (OMIM no. 601378) [31], [32], [33]. Because mutations to *CLCF1* are causal in the related syndrome CISS2, it has been broadly assumed that the central role of CRLF1 is to function as a co-ligand with CLCF1 [39], [40]. However, homozygous deletion of *Crlf1* in mice leads to perinatal lethality due to an apparent failure in suckling, indicating that complete removal of the gene is more deleterious than the loss-of-function mutations associated with CLCF1 binding and CISS1 [41]. Even though this phenotype is nearly identical to homozygous deletion of *Cntfr* in mice, it is possible that specific, cell-autonomous effects of CRLF1 are masked by premature demise of null mutants [42]. Further studies with conditional knockout alleles of *Crlf1* in the central nervous system (CNS) and skeletal muscle–another prominent site of CRLF1 expression–may provide insights into this question.

Previous studies of CRLF1 function in the mammalian CNS have primarily focused on the cellular targets of non-cell autonomous signaling through CNTFR, which include mature neurons and developing neuroblasts [43], [44], [45]. To our knowledge the precise cell type(s) that produce CRLF1 in the mammalian CNS have yet to identified, though these cells may require expression of CRLF1 even if they lack CNTFR. The cell autonomous role for CRLF1 uncovered in this study suggests that CRLF1 expression is not only important in the context of CLCF1 expression, but may also be important in cells that express CRLF1 in the absence of this binding partner or its receptor. However, it should be noted that the tumor-derived cell model system used in this study may not accurately reflect the biology of terminally differentiated, post-mitotic neurons in the mammalian nervous system, and thus should be replicated in primary cell cultures and in whole-animal models before any conclusions about potential therapeutic utility can be realized. Should these studies confirm that CRLF1 functions independent of CLCF1, it will be of significant interest to determine how this role is mechanistically executed within the cell and whether recombinant CRLF1 may be useful in neuroprotective therapies.

Future studies of CRLF1 should also address whether CRLF1 homodimers play a role in mammalian development or in adult tissue maintenance, as the binding partners for this ligand are unknown. Given the homology of CRLF1 to the extracellular ligand binding domain of other cytokine receptors, it is tempting to speculate that CRLF1 homodimers could negatively regulate other cytokines by binding and neutralizing them in the extracellular environment or within the cell. This “decoy receptor” model might explain why recombinant expression of the full-length secreted form of CRLF1 was more effective than the N-terminally truncated, non-secreted form in protecting SH-SH5Y cells from 6-OHDA toxicity, as the latter would only be able to bind cytokines prior to secretion, whereas the former would be able to bind cytokines both before and after secretion. Future studies should also address whether recombinant CRLF1 homodimers bind directly to the cell surface of SH-SY5Y cells, which would indicate the presence of receptors that could ostensibly mediate signaling by this unique molecular species.

## Supporting Information

Figure S1 **Differentiation of SH-SY5Y and SK-N-SH neuroblastoma cells fails to alter their sensitivity to mitochondrial electron transport chain inhibitors.** SH-SY5Y and SK-N-SH cells were plated to 96-well plates and differentiated either with RA-only or RA/TPA as indicated in Materials and Methods. The cells were then treated for 24 hours with the indicated mitochondrial toxins. **A-B**, Cytochrome C reductase (complex III) inhibitor antimycin A. **C-D**, ATP synthase (complex V) inhibitor oligomycin. **E-F**, Iron-sulphur cluster (complex I) inhibitor rotenone. **G-H**, Mitochondrial membrane proton gradient uncoupling ionophore FCCP (carbonyl cyanide-*p*-trifluoromethoxyphenylhydrazone).
(EPS)

Figure S2 **Culture of neuroblastoma cells in serum-free conditions induces the NF-κB signaling pathway and inflammatory gene expression.** A, Gene set enrichment analysis (GSEA) of microarray expression data indicates a strong induction of gene sets associated with the NF-κB signaling pathway (yellow highlights). **B**, Heat map of differentially expressed genes that are known targets of the NF-κB signaling pathway. **C**, Luciferase reporter assays using the 2x-κB-luc reporter vector normalized to pRL-tk-Renilla. Vectors were transfected into cells, which were then treated with the indicated media conditions as described in Materials and Methods. Fold induction values were determined relative to transfected cells cultured in NBA/10% FBS. Error bars indicate standard deviations. **D**, Survival of undifferentiated SH-SY5Y cells in response to increasing doses of 6-OHDA in the presence of different doses of interleukin-1β (IL-1β). Relative cell number was normalized to untreated cells and dose-response curves were generated as above. LD50 values ± SE are indicated in the table below the graph.
(EPS)

Figure S3 **Validation of CRLF1 shRNA vectors and effect of 6-OHDA on survival signaling pathways after CRLF1 knockdown.** A, Relative expression of CRLF1 was determined by quantitative RT-PCR in in stably selected SH-SH5Y cell lines containing control (non-targeting shRNA, NT-sh) and CRLF1-targeted shRNAs. Expression values are all shown in undifferentiated cells relative to the NT-sh control. Error bars indicate standard deviation in replicate (n = 3) samples. The two shRNAs selected for use in our study (asterix) both suppress CRLF1 expression by greater than 90%. **B**, Stably selected SH-SH5Y cell lines containing NT-sh or CRLF1-sh5 were plated to 6-well dishes and cultured either in NBA/10%FBS or for six days in RA/TPA differentiation media. The cells were then treated with the indicated doses of 6-OHDA for 1 hour, and lysates were harvested for immunoblot analysis as indicated in Materials and Methods. **C**, Stably selected SH-SH5Y cell lines containing the pCDH-EF1-IRES-neo lentiviral vector only (vector) or this vector expressing full-length (FL) or truncated (Δ34N) CRLF1 were plated to 6-well dishes and cultured either in NBA/10%FBS or for six days in RA/TPA differentiation media. The cells were then treated with the indicated doses of 6-OHDA for 1 hour, and lysates were harvested for immunoblot analysis as indicated in Materials and Methods. Data shown are immunoblots for growth/survival signaling by key pathways including the JAK/STAT (pSTAT3), MAPK (pERK), PI(3)K/AKT (pAKT) and mTOR (pS6) pathways. Total protein for STAT3, ERK1/2, AKT and S6 are included to demonstrate equal protein loading.
(EPS)

Methods S1 (DOCX)
