# Correction: Cytokine Receptor-Like Factor 1 (*CRLF1*) Protects against 6-Hydroxydopamine Toxicity Independent of the gp130/JAK Signaling Pathway

**DOI:** 10.1371/annotation/7a99f22f-8273-43c0-b78f-8675ee409bf6

**Published:** 2014-01-06

**Authors:** Brendan D. Looyenga, James Resau, Jeffrey P. MacKeigan

Several errors were introduced in the preparation of this article for publication.

There are two errors in Figure 1:

-In panel B, the bottom-most immunoblot label is incorrect. It should read β-actin.

-In panel C, the text and error bars on the chart are displaced.

Please find a correct version of Figure 1 here: http://plosone.org/corrections/pone.0066548.g001.cn.tif

There are two errors in Figure 3C:

-The error bars are displaced

-The label under the first column of the graph is incorrect. It should read IL-1β.

Please find a correct version of Figure 3 here: http://plosone.org/corrections/pone.0066548.g003.cn.tif

There are several errors in Figure 5. The "α" symbols in panels B, C and D do not display properly. Please find a correct version of Figure 5 here: http://plosone.org/corrections/pone.0066548.g005.cn.tif

There are two errors in Figure 6:

-In panels A-H, the "Δ" symbols do not display properly.

-In panels B and E, the text and error bars on chart are displaced.

Please find a correct version of Figure 6 here: http://plosone.org/corrections/pone.0066548.g006.cn.tif

