# Supplementary material for: Cytokine Receptor-Like Factor 1 (CRLF1) Protects against 6-Hydroxydopamine Toxicity Independent of the gp130/JAK Signaling Pathway
Source: PLoS One. 2013 Jun 20;8(6):e66548. doi: 10.1371/journal.pone.0066548 (PMC3688593; doi:10.1371/journal.pone.0066548)
Supplement: Methods S1 — (DOCX) [file pone.0066548.s004.docx]

***Supplemental Information***

**Cytokine receptor-like factor 1 (CRLF1) protects against 6-hydroxydopamine toxicity independent of the gp130/JAK signaling pathway**

Brendan D. Looyenga, James Resau and Jeffrey P. MacKeigan

***Supplemental Methods***

*Quantitative RT-PCR Primer Sequences*

*IL1β*: fwd (TGAACTGAAAGCTCTCCACCTCCA), rev (TTCAACACGCAGGACAGGTACAGA)

*MMP9*: fwd (ACGCAGACATCGTCATCCAGTTTG), rev (ACAACTCGTCATCGTCGAAATGGG)

*INSM2:* fwd (AAGTTTCGTCGCCAAGCCTATCTG), rev (GTGCTTCTCCCTGATATCTGCTGT)

*PXDNL*: fwd (ACCCAGTTTCAGCGGCTAAGAGAT), rev (TGTCACCATTGTCACAAAGCACCC)

*CRLF1*: fwd (AACATCAGCTGCTGGTCCAAGAAC), rev (TGTTGTCCTGGCCATACCACCTAA)

*RPL13A*: fwd (TAAACAGGTACTGCTGGGCCGGAA), rev (AAGGGTTGGTGTTCATCCGCTT)

*Luciferase reporter assays*

SH-SY5Y and SK-N-SH cells were plated to 24-well plates at a density of 5.0 x 10^4^ cells/well and allowed to adhere overnight. The next day they were transfected with 150 ng/well of 2x-κB-luc reporter plasmid and 10 ng/well of pRL-tk-Renilla control plasmid using Fugene HD lipid transfection reagent (Roche, Penzberg, Germany) in serum-free OptiMEM media. 24 hours after transfection the media was removed and replaced with NBA containing 10%FBS, or serum-free media (NBA/B27 Supplement) containing RA (10 µM) or TPA (100 nM). Cells were cultured for three days, and then media was replaced for additional three days with the same media conditions. Lysates were harvested with passive lysis buffer and analyzed for firefly and Renilla luciferase expression using the dual luciferase reporter assay (Promega). Raw luminescence values were obtained using an Envision plate reader (Perkin-Elmer). The 2x-κB-luc reporter values were divided by constitutive pRL-tk-Renilla expression (firefly RLU/ Renilla RLU) to obtain normalized activation of the NF- κB signaling pathway in each condition.
